# Recording fine‐scale movement of ground beetles by two methods: Potentials and methodological pitfalls

**DOI:** 10.1002/ece3.7670

**Published:** 2021-05-16

**Authors:** Jana Růžičková, Zoltán Elek

**Affiliations:** ^1^ MTA‐ELTE‐MTM Ecology Research Group Biological Institute, Eötvös Loránd University Budapest Hungary

**Keywords:** azimuth, carabids, compass, distance‐bearing, global positioning system, state‐switching models

## Abstract

Movement trajectories are usually recorded as a sequence of discrete movement events described by two parameters: step length (distance) and turning angle (bearing). One of the most widespread methods to record the geocoordinates of each step is by a GPS device. Such devices have limited suitability for recording fine movements of species with low dispersal ability including flightless carabid beetles at small spatio‐temporal scales. As an alternative, the distance‐bearing approach can avoid the measurement error of GPS units since it uses directly measured distances and compass azimuths. As no quantification of measurement error between distance‐bearing and GPS approaches exists so far, we generated artificial fine‐scale trajectories and in addition radio‐tracked living carabids in a temperate forest and recorded each movement step by both methods. Trajectories obtained from distance‐bearing were compared to those obtained by a GPS device in terms of movement parameters. Consequently, both types of trajectories were segmented by state‐switching modeling into two distinct movement stages typical for carabids: random walk and directed movement. We found that the measurement error of GPS compared to distance‐bearing was 1.878 m (*SEM* = 0.181 m) for distances and 31.330° (*SEM* = 2.066°) for bearings. Moreover, these errors increased under dense forest canopy and rainy weather. Distance error did not change with increasing distance recorded by distance‐bearing but bearings were significantly more sensitive to error at short distances. State‐switching models showed only slight, not significant, differences in movement states between the two methods in favor of the random walk in the distance‐bearing approach. However, the shape of the GPS‐measured trajectories considerably differed from those recorded by distance‐bearing caused especially by bearing error at short distances. Our study showed that distance‐bearing could be more appropriate for recording movement steps not only of ground‐dwelling beetles but also other small animals at fine spatio‐temporal scales.

## Graphical abstract

In this study, we compared two different approaches for recording the fine‐scale movement of ground‐dwelling beetles: distance‐bearing and GPS. We found that the measurement error of a GPS device at short distances up to 20 m considerably modified the shapes of recorded trajectories which may bias the real biological signal.

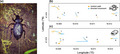

## 1 INTRODUCTION

Animal movement is one of the fundamental aspects of behavioral ecology. A path made by a moving individual is continuous, yet it is routinely recorded as a sequence of discrete movement steps (Turchin et al., 1991). Each step, as the position of an individual in space and time, has two major components: a step length (distance) and a turning angle (bearing) between consecutive steps (Calenge et al., 2009; Kareiva & Shigesada, 1983). Reliable estimates of these two parameters are essential for understanding the distribution of individuals in their environments (Holyoak et al., 2008; Nathan et al., 2008). One of the most popular approaches to collect movement data is to attach very high frequency (VHF) or Global Positioning System (GPS) transmitters to individuals and record their positions at regular time intervals (Cagnacci et al., 2010; White & Garrott, 1990). The movement path is then recorded as a sequence of geocoordinates or marked on a high‐resolution georeferenced map (White & Garrott, 1990). Although many types of GPS devices are available for scientific work (e.g., Negro et al., 2017; Rink & Sinsch, 2007), the positional imprecision of these devices can add measurement errors to recorded geocoordinates (Adrados et al., 2002; Bradshaw et al., 2007; Frair et al., 2010). The accuracy of GPS devices can be influenced by several factors, including signal jamming by satellite position (Ranacher et al., 2016), atmospheric interference (Frair et al., 2010), topographic complexity, the density of canopy cover (D'Eon et al., 2002), and the device own measurement error (Ranacher et al., 2016).

The majority of movement ecology studies are focused on the movement of large animals, such as deers, wild horses, or wolves (e.g., Millspaugh & Marzluff, 2001; White & Garrott, 1990), where spatial scales can range from hundreds of meters to kilometers and temporal scales from days to years. Nevertheless, tracking periods of smaller animals, especially ground‐dwelling insects, are limited only to a period of a few days or weeks due to the limited battery life of VHF transmitters. To maximize the cost/benefit ratio in obtaining a sufficient amount of high‐resolution movement data, ground‐dwelling insects are tracked as often as possible, usually every few hours. Covered distances per one step barely exceed a few tens of meters, and these studies focus on daily movement patterns within and between small habitat patches (e.g., Negro et al., 2017; Riecken & Raths, 1996; Růžičková & Veselý, 2018). Nevertheless, the estimated positions of commercially available GPS devices (to record exact coordinates) are relatively accurate above 3 m distance but below that distance, the measurement error progressively increases (Ranacher et al., 2016). As this is the range usually covered daily by walking insects, the measurement error can add a bias when a trajectory is described/recorded by a GPS device; hitherto, this issue is routinely ignored in movement ecology studies for insects (but see Fernández et al., 2016).

Although several methods are available for recording animal paths without remote‐sensing technique, their utilization requires experience for the correct estimation of parameters of each movement step (Fisher et al., 2020; Turchin et al., 1991). The “coordinate geometry” method, also called the distance‐bearing method, can be an ideal option to avoid the bias of the GPS method at fine‐scale mapping (Turchin et al., 1991). In distance‐bearing (DB), the coordinates of the target location are obtained from the last known position by using the directly measured distance and the compass azimuth (bearing; Baars, 1979; Robinson et al., 2020; Wallin & Ekbom, 1988). This method has a long tradition for recording paths of insects at fine spatio‐temporal scales; nevertheless, it has widely been used only in studies of butterflies as they can be relatively easily followed due to their conspicuous flying behavior (e.g., Fisher et al., 2020; Schultz & Crone, 2001; Skórka et al., 2013). In studies on the movement ecology of other insect groups, including ground‐dwelling beetles, distance‐bearing has largely been abandoned due to the rapid development of easy‐to‐use hand‐held GPS devices (but see Růžičková & Veselý, 2018).

Although fine‐scale movement maps generated by using either distance‐bearing or GPS can differ, no quantification of such difference exists so far. Inaccurate positional data might completely mask a biological signal extracted from the movement path, such as microhabitat resource use (Bradshaw et al., 2007). Habitats are not homogeneous but consist of a spatial mosaic of different microhabitat patches. For instance, the distribution of rocks, bare soil, dead woody debris, leaf litter, or shrubs changes within a few meters in the managed temperate forest (Elek et al., 2018; Negro et al., 2017). These patches can have different functions, serving as shelters, overwintering, oviposition, or foraging sites, and ground‐dwelling species show a nonrandom distribution associated with a certain microhabitat (Niemelä et al., 1992; Pearce et al., 2003; Wehnert & Wagner, 2019). Thus, it is important to be able to precisely describe microhabitat selection at fine spatial scales based on individual movement for understanding how a particular species persists in its environment and consequently adjust possible management (Negro et al., 2014).

In this study, we compared GPS and distance‐bearing approaches in recording fine‐scale movement in two experiments where we considered simulated as well as actual trajectories made by ground‐dwelling insects. As a model group, we selected ground beetles (Coleoptera: Carabidae), one of the most frequently radio‐tracked insect groups. In the first experiment, we artificially generated trajectories that were based on movement parameters derived from already existing movement data for large species of genus *Carabus*. As their step length between relocations rarely exceeds 20 m within few hours (e.g., Růžičková & Veselý, 2018), we supposed that the measurement error up to this distance recorded by distance‐bearing is lower than that of a GPS device. Therefore, we considered locations (hereafter fixes) obtained by distance‐bearing as control and those recorded by the GPS method as biased ones, that is, the measurement error represents the error of a GPS device. In the second experiment, we employed radio telemetry and tracked living specimens of *Carabus coriaceus* equipped by small VHF transmitters in an oak‐hornbeam forest. We recorded their fixes every four hours by both methods and hypothesized that additional factors interfering with GPS‐signal, such as dense forest canopy and weather, could increase the measurement error and notably affect recorded movement parameters and trajectory profiles.

In particular, we focused on two major research questions: (1) Is there any significant difference in the magnitude of measurement error between DB‐measured and GPS‐measured trajectories regarding distances and bearings? (2) If so, how can the measurement error bias a trajectory shape and consequent biological signal represented by the two movement patterns typical for carabid beetles, a random walk and a directed movement, based on state‐switching model estimates?

## 2 MATERIAL AND METHODS

### 2.1 Artificial trajectories and their record by DB and GPS

The first experiment was conducted with artificially generated fine‐scale trajectories. Each generated trajectory was represented as a sequence of discrete consecutive fixes where each fix was defined by two parameters: step length (distance) and turning angle (bearing) between successive movements (Calenge et al., 2009; Kareiva & Shigesada, 1983; Marsh & Jones, 1988). These parameters were generated in R 3.6.1 (R Core Team, 2019) for each fix separately in the following way:

1. The step length in meters was selected in a four‐step randomization process. First, we generated 100 random numbers between 0.5 and 20.0 using the *runif* function rounded to one decimal number. Minimal and maximal values corresponded with distances (in meters) usually covered by large *Carabus* species per one fix (existing data for *C. coriaceus*: Riecken & Raths, 1996; *C. hungaricus*: Bérces & Růžičková, 2019; *C. olympiae*: Negro et al., 2008; Negro et al., 2017; *C. ullrichii*: Růžičková & Veselý, 2016, 2018). From this random set, we sampled 50 values using the *sample* function without replacement, and then five values were selected by the same function. As the final step, we randomly selected one value out of five by ourselves for the step length to avoid the consistent error added by randomization by computers.
2. The turning angle, an absolute turning angle toward a fixed point, here as the magnetic north, was selected in the same process as in the step length. As a primary random set, 100 integers between 1 and 12 were generated using the *runif* function following a uniform distribution. Here, 1 corresponded with 30° compass azimuth and 12 with 360/0° azimuth (i.e., toward the north), thus having 12 discrete units per 30°. This randomization procedure helps to avoid any autocorrelation in turning angles, which can distort the further simulations (Dray et al., 2010). The whole selection process was repeated until we generated three trajectories with 10 fixes and three trajectories with 20 fixes. As was already demonstrated by Turchin et al. (1991), this number of fixes per trajectory was sufficient to get an adequate description of individual movement.

**FIGURE 1:**
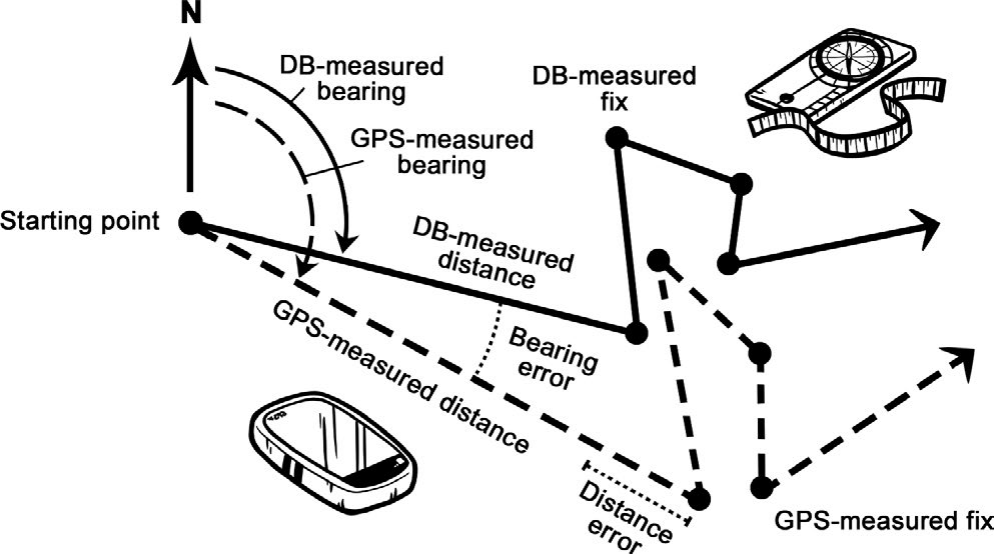
Overview of two methods, distance‐bearing (DB) and GPS, and their expected differences in fix recording. DB‐measured trajectory (solid line) was recorded by magnetic compass with a protractor and measuring tape, while fixes of GPS‐measured trajectory (dashed line) were recorded by GPS device. Due to measurement errors in distances and bearings (dotted line) of the employed GPS device, GPS‐measured trajectory differs from DB‐measured one. For better visibility, the movement parameters including error terms are shown only between the first two fixes, but in fact they were measured for every fix

The experimental site was grassland with a few solitary trees to avoid possible additional GPS measurement errors due to dense vegetation and slope (Frair et al., 2010). The site was situated in the outskirt of Budapest, Hungary, on the western bank of the river Danube (47.4724°N, 19.0609°E). Approximately in the middle of the experimental area, we randomly chose a point of reference and its geolocation was recorded by a hand‐held GPS device (Garmin Dakota 20, in WGS84 coordinate reference system, accuracy ~3 m). From this point, we manually built the trajectory (with predefined fixes generated above) using a measuring tape (0.3 cm accuracy) and a magnetic compass (1° accuracy) with a protractor. The GPS coordinates for each fix were recorded by the above‐mentioned device. Only a single GPS logger was used. Thus, distance‐bearing was used to build a particular trajectory, and then, its fixes were GPS‐measured. The relationship between DB‐measured and GPS‐measured trajectories and measurement errors is described in Figure 1. Magnetic declination was considered as an angular difference between the magnetic north (the direction of the compass needle) and the true geographic north. Its estimated value for the experimental site was +5.133°, that is, toward the east (World Magnetic Model, www.ngdc.noaa.gov/geomag/WMM, accessed 20 April 2020). Each of the six trajectories was built three times de novo from the same starting point with at least one‐day break between builds. All GPS coordinates were sampled only during sunny days with max. 20% cloud coverage to avoid any additional atmospheric interference. To sum up, we collected 384 fixes in 24 trajectories; six DB‐measured, and 18 (three repeats of six) GPS‐measured tracks. Data sampling was conducted between April and June 2020.

### 2.2 Radio tracking of living carabids

In the second experiment, extra GPS‐signal interfering factors, such as dense canopy cover and weather, were added into the study system. We used living specimens of the large (body size of 33–40 mm) carabid species *Carabus coriaceus,* commonly occurring in temperate forests of Hungary. Six individuals (three males and three females) were equipped with small VHF transmitters (the PicoPip model, weight of 0.29 g, manufactured by Biotrack Ltd, Wareham, UK; Figure 4a) attached on their elytra by super glue. The tag/body mass ratio was approximately 14%. We presumed that tagging did not substantially disturb beetles’ walking behavior; nevertheless, the true impact of tracking devices on insects mostly remains unknown and has to be critically evaluated (Batsleer et al., 2020). Tagged beetles were released at least 20 m apart from each other in a temperate oak‐hornbeam forest in the Pilis Mountains, northern Hungary (47.6741°N, 18.9105°E) and manually tracked every four hours (i.e., six times per day) for five consecutive days in September 2018. As tracking equipment, we used a hand‐held Sika receiver with Yagi directional and 20 cm dipole antennas.

The primary study on the movement activity of *C. coriaceus* (Elek et al., 2019) focused on species’ habitat use and the impact of forest management on individual activity; thus, all details about the ecological context can be found there. In this experiment, we extended the methodological part concerning fix recording. All tracked beetles were localized by a so‐called “homing procedure” (White & Garrott, 1990): We started the searching for the tagged beetle at its last known position (i.e., previous fix; marked by a wooden pole set into the ground) and then followed the transmitter's signal up to a distance of 0.5 m from the expected signal source to secure the tagged beetle will not be crushed under food (Riecken & Raths, 1996). Then, the new fix was recorded by both methods: distance‐bearing (using a compass and a measuring tape), as well as the GPS device (Garmin Dakota 20, i.e., the same model as in the experiment with the artificial trajectories), and another wooden pole was installed. If the covered distance was lower than 0.5 m, the fix was considered as passive, that is, with no activity. In total, we collected 320 fixes in 12 trajectories (six recorded by distance‐bearing and six by GPS).

### 2.3 Data analyses

The same statistical approach was used in both experiments. To compare DB‐measured and GPS‐measured fixes, first, we had to obtain step lengths and bearings from GPS‐measured coordinates. We used the *as.ltraj* function from the “AdehabitatLT” package which can compute several descriptive parameters of the trajectory from a set of GPS coordinates (see Calenge, 2006 and Calenge et al., 2009 for details). In the created *ltraj* object, we looked specifically for two parameters: distance (dist) between successive fixes and absolute angle (abs.angle) between x‐direction (longitude in this case) and the step direction (see Marsh & Jones, 1988 for details). The latter parameter is originally provided in radians and with a different orientation than in the DB approach, so we converted it to compass azimuths where absolute angle 0 rad corresponded to 90° azimuth (east), +π/2 rad to 0° (north), ‐π/2 rad to 180° (south), and finally π rad to 270° (west, Marsh & Jones, 1988). Second, we obtained long/lat coordinates from DB‐measured fixes using Azimuth and Distance Plugin (de Paulo et al., 2019) in QGIS 3.4.2 (Version “Madeira,” QGIS Development Team, 2019). The plugin can draw a trajectory from a list of distances and compass azimuths. As a starting point, we used GPS coordinates of the reference point (in the first experiment) or beetles’ releasing point (in the radio‐tracking experiment) to concatenate the DB‐measured trajectory in long/lat coordinate system and to ensure that the corresponding pair of trajectories (DB‐measured and GPS‐measured) starts from the same point. When the trajectory was drawn, we extracted its long/lat coordinates.

The correlation between DB‐ and GPS‐measured long/lat coordinates was quantified by Pearson correlation. We calculated differences (measurement errors) between DB‐ and GPS‐measured distances and bearings for the corresponding pair of fixes (Figure 1). If no measurement error existed, the difference was zero. For distance error, the positive values indicated larger GPS‐measured distances than DB‐measured ones and vice versa if negative. In the case of bearings, absolute values were used due to the circular nature of the variable. To test the relation of both types of errors to DB‐measured distances and bearings and whether these errors can be affected by fix order, we used linear mixed models with a normal distribution and identity link function (the *lmer* function from the “lme4” package, Bates et al., 2015). In a single‐argument model, DB‐measured distances, bearings, the fix order in the trajectory were considered as a fixed effect and the track ID as a random effect. We also tested whether the total length of the trajectory (i.e., the sum of all step lengths) differs between recording methods. The explanatory power of each model was tested by marginal R^2^ for fixed effects and conditional R^2^ for random effects using the *r.squaredGLMM* function from the “MuMIn” package (Bartoń, 2016). In the radio‐tracking experiment, carabid beetles often exhibited no activity between consecutive fixes (sensu Bérces & Růžičková, 2019; Riecken & Raths, 1996; Růžičková & Veselý, 2018 for other species). These passive fixes were excluded from linear mixed models as measurement errors in distances and bearings could be calculated only from active fixes.

For segmenting both DB‐ and GPS‐measured trajectories into distinct movement states, we used hidden Markov models (HMMs; Michelot et al., 2016). In carabids, two distinct movement stages can be distinguished. A *random walk* is described by short step length and high variation in bearings; this is characteristic for foraging behavior and the presence of suitable habitat (Baars, 1979). Long step distances with the same turning angles are characteristic of a *directed movement* that usually results as an indented migration from one habitat to another (Baars, 1979; Kareiva & Shigesada, 1983). For a two‐state trajectory segmenting, we first fitted the HMM only on the DB‐measured trajectories using the *fitHMM* function from the “moveHMM” package (Michelot et al., 2016) to ensure the optimal decoding of the random walk and the directed movement. As initial values of the model parameters, the mean step length was set at 1 m for the random walk and 10 m for the directed movement, respectively. Since the gamma distribution was used to model step length, we set the initial *SD* of the same order as the mean value of a particular movement state. The mean turning angle was *π* for the random walk with an angle concentration of 0.5. The concentration parameter close to zero corresponds with a uniform distribution of turning angles (i.e., undirected movement); the higher it gets, the more directed movement is. For the directed movement, the mean turning angle was set at 0 rad with the concentration of 2.5 (see Michelot et al., 2016 and package's vignettes for details). For the radio‐tracking experiment, zero inflation was specified as 0.5 for both movement states. Subsequently, the model with the same parametrization was fitted to GPS‐measured trajectories. Under the fitted HMM, each trajectory was decoded by the Viterbi algorithm into the sequence of two movement states and plotted as a trajectory map where each fix was colored according to a particular movement state. We counted the number of random walks and directed movements for each trajectory. Generalized linear mixed model (the *glmer* function) with a binomial distribution and logit link function was used to test whether movement states (represented as a proportion of random walk) differed between DB‐measured and GPS‐measured trajectories. The response was coded as a two‐column matrix of [random walk, directed movement] using the *cbind* function (Grueber et al., 2011). In the model, the recording method (DB versus GPS) was a fixed effect and a track ID was a random effect.

## 3 RESULTS

### 3.1 Artificial trajectories

**FIGURE 2:**
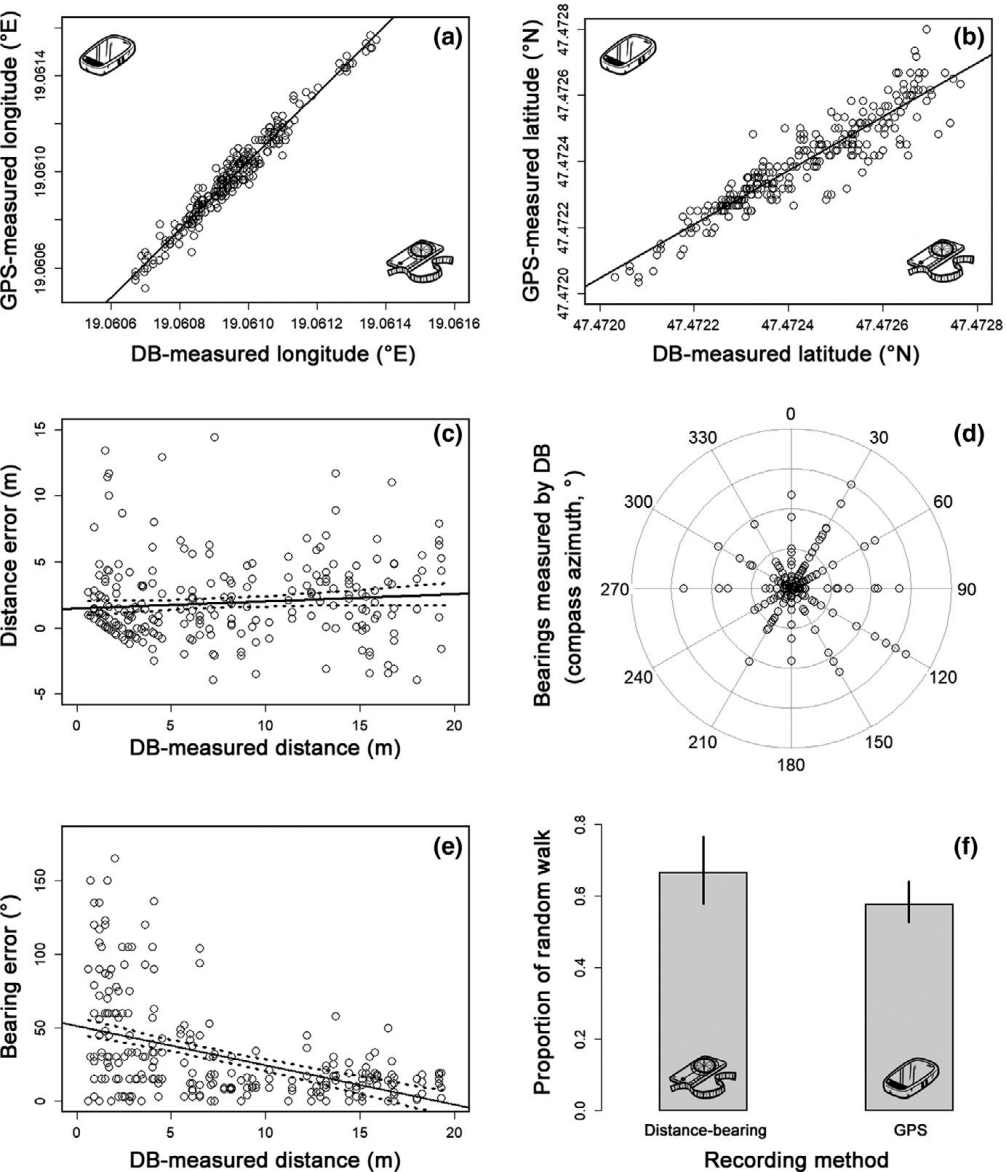
The correlation between DB‐measured and GPS‐measured longitude (a) and latitude (b) coordinates from the experiment with artificial trajectories. The relationship between DB‐measures distances (c), bearings (d), and their errors; and between bearing errors and DB‐measured distances (e). The concentric circles in (d) represent the magnitude of bearing errors. The proportion of random walk as one of the movement states in a particular recording method is shown on (f). Dashed lines and whiskers represent a 95% confidence interval

In total, we collected 384 fixes, including starting points, for 24 trajectories; six DB‐measured, and 18 (three repeats of six) GPS‐measured tracks. The comparison of DB‐measured and GPS‐measured long/lat coordinates showed a strong correlation in the longitude (Pearson *r* = 0.981, *t* = 84.815, *df* = 267, *p* < .001, Figure 2a) but less in the latitude (Pearson *r* = 0.929, *t* = 41.007, *df* = 267, *p* < .001, Figure 2b) suggesting greater measurement error in latitudinal coordinates.

Distances recorded by GPS device were on average 1.878 m (*SEM* = 0.181 m) larger than the DB‐measured ones, and the size of the measurement error did not change with increasing covered distance (Figure 2c). For the total length of the trajectory, the trajectories recorded by GPS were significantly longer than the distance‐bearing ones by about 28.1 m (Tables 1 and 2). The average bearing error of GPS was 31.330° (*SEM* = 2.066°), and it was evenly distributed among all directions (Figure 2d). However, the size of the bearings error was significantly higher at shorter than longer distances (Table 2, Figure 2e). The number of fixes recorded in a trajectory did not have an effect either on distance error or on bearing error.

**TABLE 1:** Comparison of fine‐scale trajectories recorded by two methods: distance‐bearing and GPS from the experiment with artificial trajectories (a) and radio tracking of six *Carabus coriaceus* individuals (b)

ID	No. of fixes	Distance‐bearing		GPS	
		Total length (m)	Step length (m)	Total length (m)	Step length (m)
(a)					
1	10	85.0	8.5 (1.2; 18.3)	104.3	10.4 (1.6; 23.8)
2	10	88.1	8.8 (1.5; 19.2)	117.7	11.9 (2.4; 25.8)
3	10	66.3	6.6 (0.9; 15.1)	78.3	7.9 (1.6; 18.4)
4	20	150.0	7.5 (0.7; 19.3)	184.9	9.3 (1.6; 27.7)
5	20	134.6	6.7 (0.6; 16.8)	153.8	7.7 (1.6; 21.7)
6	20	144.1	7.2 (0.9; 19.2)	193.9	9.7 (1.6; 25.4)
(b)					
1	11	65.4	5.9 (0.5; 14.7)	105.3	9.6 (2.2; 24.0)
2	4	25.4	6.4 (0.5; 19.8)	35.9	9.0 (2.4; 21.4)
3	4	8.0	2.0 (0.5; 4.7)	22.9	5.7 (3.7; 9.4)
4	4	33.9	8.5 (1.2; 23.1)	89.2	22.3 (1.5; 48.1)
5	3	3.9	1.3 (0.5; 1.8)	14.6	4.9 (2.4; 6.9)
6	6	43.3	7.2 (1; 21.3)	40.0	6.7 (1.9; 18.16)

Note

Total length of the trajectory is a sum of all step lengths in a particular trajectory. Step length represents the mean (minimum; maximum) distance covered in one movement step. The number of fixes in radio‐tracking experiment shows only active fixes with recorded movement activity of tagged beetles. Trajectory IDs follow the same numbering as in ESM Figures S1 and 4.

**TABLE 2:** The results of performed models from the experiment with artificial trajectories, the significant effects are in bold

Models	*χ* ^2^	*df*	*p*	Marginal *R* ^2^	Conditional *R* ^2^
Distance error ~ DB‐measured distance	2.702	1	.100	0.009	0.085
Bearing error ~ DB‐measured bearing	14.254	11	.219	0.049	0.075
Bearing error ~ DB‐measured distance	**74.684**	**1**	**<.001**	**0.211**	**0.245**
Distance error ~ fix order	0.218	1	.641	0.001	0.079
Bearing error ~ fix order	1.469	1	.226	0.005	0.022
Total trajectory length ~ recording method	**16.946**	**1**	**<.001**	**0.070**	**0.905**
Proportion of random walk ~ recording method	2.413	1	.120	0.009	0.009

Note

The response variables “distance error” and “bearing error” were based on the differences between GPS‐measured and DB‐measured movement parameters. Model explanatory power was tested by marginal *R* ^2^ for fixed effects and conditional *R* ^2^ for random effects (here as a track ID).

The visual inspection of plotted trajectories revealed large differences in shapes between DB‐ and GPS‐measured ones (ESM Figures S1), mostly due to bearing error at short distances (see above). HMMs revealed that the proportion of random walk in DB‐measured trajectories was 8.89% higher than in those recorded by GPS device but this effect was not significant (Table 2, Figure 2f); in other words, slightly more steps were described as directed movement in GPS‐measured trajectories. The transition probability between movement states was 48.35% from random walk to directed movement and 69.48% in the opposite direction.

### 3.2 Radio tracking of *Carabus coriaceus*

**FIGURE 3:**
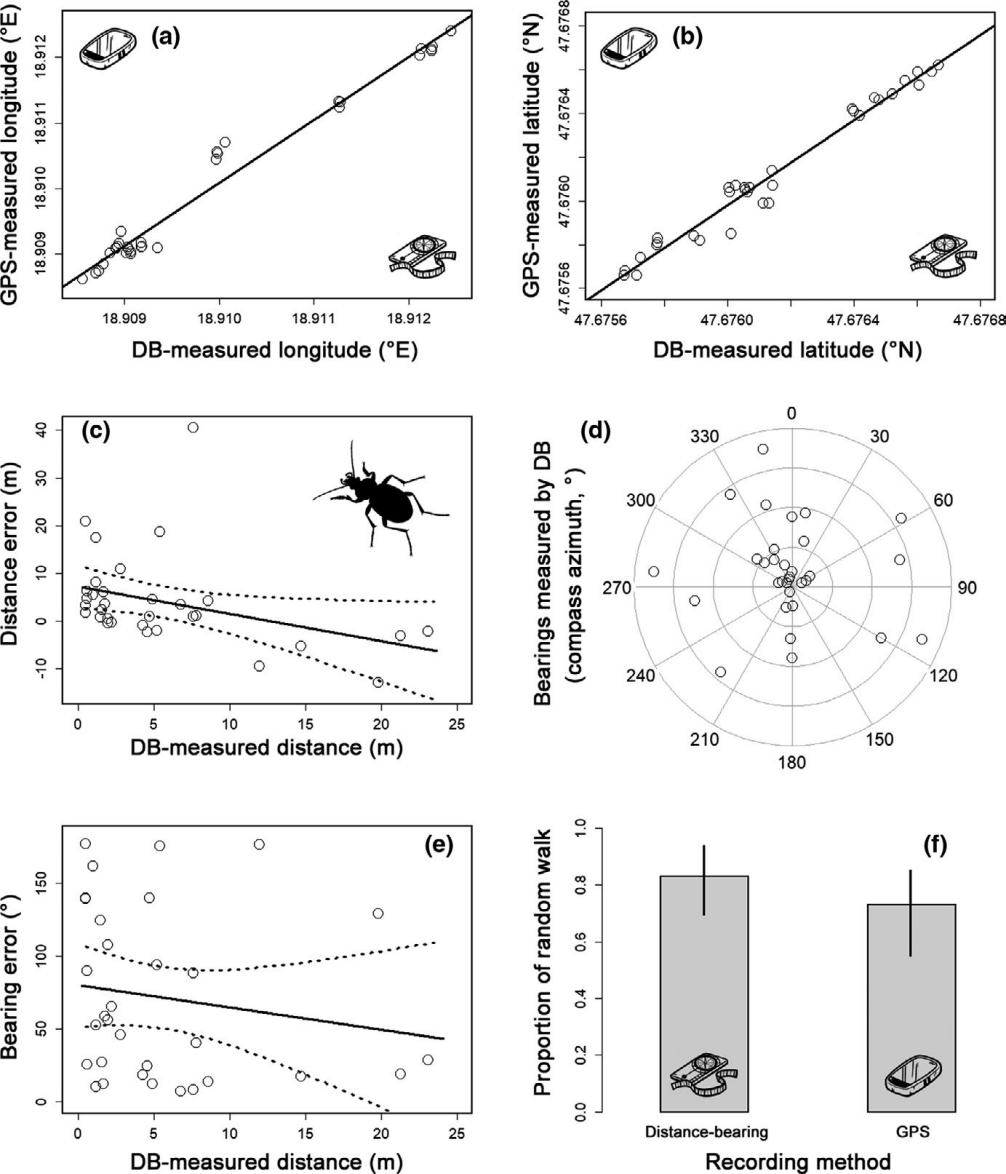
An experiment with fine‐scale radio tracking of *Carabus coriaceus* in a temperate forest illustrating the impact of the measurement error on movement parameters recorded by GPS device. The comparison of DB‐measured and GPS‐measured longitude (a) and latitude (b) coordinates showed their strong correlation. Regarding movement parameters, the relationship between DB‐measured distances (c), bearings (d), and their errors are displayed as well as between bearing error and DB‐measured distances (e). In (d), the concentric circles represent the magnitude of bearing error. The proportion of the random walk as one of the movement states is shown on (f). Dashed lines and whiskers represent a 95% confidence interval

**FIGURE 4:**
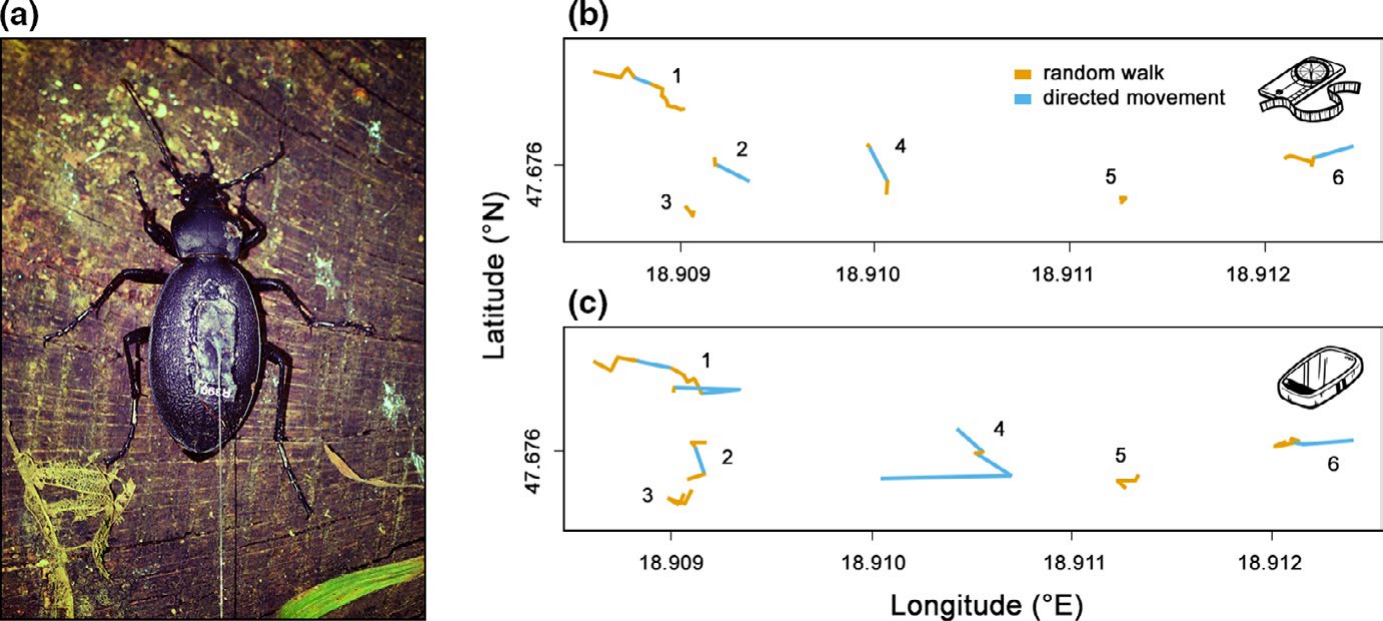
Female of *Carabus coriaceus* with attached radio transmitter (a). Trajectories (1–6) decoded by the Viterbi algorithm into two movement states noticeably differed in their shapes between DB (b) and GPS (c) recording methods

Radio tracking of six *Carabus coriaceus* individuals (Figure 4a) in the forest gave us, in total, 320 fixes, including releasing points, 160 for a particular recording method. Due to behavioral constrains of the species, only 64 fixes (32 per method) were with activity (Table 1). We found a strong correlation between DB‐ and GPS‐measured longitude (Pearson *r* = 0.987, *t* = 33.230, *df* = 30, *p* < .001, Figure 3a) as well as latitude (Pearson *r* = 0.985, *t* = 31.809, *df* = 30, *p* < .001, Figure 3b).

Distances recorded by GPS were on average 3.992 m (*SEM* = 1.718 m) larger than those recorded by distance‐bearing and distance error significantly decreased with increasing covered distance (Table 3, Figure 3c). The mean bearing error was 71.639° (*SEM* = 10.281°), and it was evenly distributed to all directions (Figure 3d). Bearing error slightly decreased toward larger distances but this trend was not significant (Table 3, Figure 3e). The total length of trajectory was on average 21.3 m longer when recorded by GPS than distance‐bearing (Tables 1 and 3).

**TABLE 3:** The results of performed models from the radio‐tracking experiment, the significant effects are in bold

Models	*χ* ^2^	*df*	*p*	Marginal *R* ^2^	Conditional *R* ^2^
Distance error ~ DB‐measured distance	**5.833**	**1**	.**016**	**0.146**	**0.263**
Bearing error ~ DB‐measured distance	0.815	1	.366	0.025	0.041
Total trajectory length ~ recording method	**5.744**	**1**	.**017**	**0.116**	**0.779**
Proportion of random walk ~ recording method	1.551	1	.213	0.044	0.219

Note

The response variables “distance error” and “bearing error” were based on the differences between GPS‐measured and DB‐measured movement parameters. Models explanatory power was tested by marginal *R* ^2^ for fixed effects and conditional *R* ^2^ for random effects (here as a track ID).

Concerning movement patterns, the proportion of the random walk was slightly but not significantly higher when trajectory was recorded by distance‐bearing than GPS (Table 3, Figure 3f). However, the measurement error, especially at short distances, notably disturbed trajectory profiles resulting in completely different profiles (Figure 4b and c).

## 4 DISCUSSION

Obtaining precise positional data is challenging, especially in fine‐scale movements of walking insects including carabids. We simulated as well as followed their movement and showed that fixes recorded by a GPS device considerably differed from those measured by distance‐bearing due to errors in both distances and bearings leading to different trajectory shapes. Moreover, this measurement error was higher in the radio‐tracking experiment likely due to dense canopy cover and rainy weather. Rather than using a high‐precision professional GPS logger, we collected all geocoordinates by a commercially available device. These units are commonly utilized by researchers in the field and likely have a similar magnitude of the measurement error (e.g., Fernández et al., 2016; Ranacher et al., 2016; Růžičková & Veselý, 2016). Therefore, our findings may help the design especially the methodological aspects of future studies focused on movement in different habitats at the fine spatio‐temporal scale. We highlight that the distance‐bearing method for recording fixes may overcome the problem with the measurement error of GPS devices, especially at short distances in the order of a few meters.

### 4.1 The magnitude of measurement error

In the experiment with artificial trajectories, we found that the measurement error in the distance was nearly 2 m and did not increase with greater distance covered. This contradicts the results of Ranacher et al. (2016) who found that overestimation of GPS‐measured distances tended to rise with increasing distances due to low spatial autocorrelation of distant locations. Previously, only Fernández et al. (2016) tested the reliability of recorded positions in the fine‐scale movement of butterflies and reported the mean distance error as 0.033 m, with a maximum value of 0.25 m for a similar, hand‐held GPS device. Their values were a magnitude lower than ours, probably because of the different methods of quantification. The authors used coordinates of easily recognizable landmarks, such as solitary trees or shrubs, for calibrating butterfly positions. Benchmarking is based on an already known fixed location and usually produces measurement error below 1 m (Robinson et al., 2020). Contrarily, geocoordinates obtained from moving large animals without any reference position may suffer from relatively high measurement error which, in some cases, can exceed 100 m (Frair et al., 2010; Montgomery et al., 2010). Although these studies were published a decade ago and the technological advances made the precision of GPS devices better since then, the current measurement error still remains at ≤3 m (Ranacher et al., 2016). It seems to be possible to overlook errors in the distance in flying, mobile species with high dispersal power (e.g., butterflies, dragonflies, flying beetles) where the one movement step exceeds one hundred meters (Rink & Sinsch, 2007), but not in ground‐dwelling insects that cover much less distance on the ground. Several *Carabus* species cover only a few meters between two tracking sessions at the temporal scale of a couple of hours (Negro et al., 2017; Riecken & Raths, 1996; Růžičková & Veselý, 2018). As the distance recorded by GPS device is higher due to measurement error, it leads to the overestimation of total distance in movement paths. This can be an issue if GPS‐measured distances are used for dispersal estimations. The overestimated dispersal capacity of target species may lead to ineffective management practices (e.g., forming corridors or stepping stones in fragmented landscapes), reducing species ability to disperse between suitable (micro)habitat patches as the distance between them is too large to cover. Hitherto, if the tagged individual did not move between two tracking sessions as ground beetles frequently do (see Bérces & Růžičková, 2019; Riecken & Raths, 1996), but the geocoordinates are recorded for both events, the measurement error may indicate false movements.

The bearing error was approximately 31° from the estimated directions. However, it was significantly higher at short distances, resulting in completely different shapes of trajectories. For GPS collars, Jerde and Visscher (2005) reported the same issue at the large spatial scale where bearings were accurate only when the observed distance between two fixes was large in relation to the measurement error of a GPS device. They suggested re‐scaling the temporal resolution of sampling to increase the distance covered by tracked individuals between consequent measurements, for example, by obtaining a fix only every 2 hr instead of every 5 min (Jerde & Visscher, 2005). The proposed solution is, however, not suitable for ground‐dwelling beetles due to the fine temporal scale. Usually, they are tracked every few hours to obtain a high resolution of movement data. It can take several days until the tracked individual walks far enough to overcome the measurement error of the employed GPS device. Moreover, further reduction of sampling resolution is not possible due to the short battery life of used VHF transmitters which is a couple of weeks at maximum.

### 4.2 The possible bias of the biological signal and additional interfering factors

Shapes of GPS‐measured trajectories differed substantially from those recorded by distance‐bearing in both experiments. HMMs showed a slight underestimation of random walks in the GPS‐measured trajectories. The measurement error can increase in rainy weather as well as in other habitat types, especially in densely overgrown vegetation, where the closed (and occasionally wet) canopy acts as a strong interfering factor for GPS signals (Frair et al., 2010). A higher measurement error may bias a trajectory profile, and consequently, state‐space modeling may result in a higher proportion of directed movement due to greater distances and incorrect bearings (see Bradshaw et al., 2007 for a large‐scale case study). Indeed, our radio‐tracking experiment conducted in the temperate forest showed even higher measurement error either in distance (4 m error) or bearings (72° error) than in the experiment with artificial trajectories due to the dense canopy and atmospheric interferences (we tracked beetles regardless of weather conditions from sunny days to rain). As a result, the trajectories recorded by a GPS device were notably different in shape than those recorded by distance‐bearing.

For manual tracking, usually, the so‐called “homing” procedure is implemented (White & Garrott, 1990). It requires being as close as possible to the tracked individual to record its exact position. Therefore, it should be noted that homing of ground‐dwelling beetles can potentially increase the risk of crushing them under the foot or elicit their escape behavior and consequently flawed recorded trajectories. There is no such disturbance in GPS transmitters. However, the lightest available GPS tags weigh more than 3.5 g (Lotek Wireless Inc., accessed 12 March 2021) making these devices not eligible for tracking insect movement. To minimize the possible observer‐induced disturbance, Riecken and Raths (1996) suggested stopping manual tracking at a 0.5 m distance from the expected signal source. Based on our radio‐tracking experiences, only once we hit the situation that tracked beetle walked in front of us. In this case, we recorded the fix at the spot where tracked beetle was observed for the first time to avoid additional disturbances caused by following it. In the rest of the tracking sessions, tagged individuals were hidden in the forest litter or under the ground; thus, our presence nearby likely was not disturbing.

Moreover, we want to mention one potential computational bias by the software used for movement analyses that may also add extra error to the dataset. It seems the R package “AdehabitatLT” (Calenge et al., 2009) had a preset value for minimum detected distance as 1.6 m. When the estimated distance is less than 1.6 m, the *as.ltraj* function automatically rounds it up to this value. Thus, it is not possible to estimate the movement distances under 1.6 m.

## 5 CONCLUSION

In this study, we presented and discussed several issues of using a GPS device for recording movements of ground‐dwelling beetles at the fine spatio‐temporal scale. We can conclude that the distance‐bearing method is more appropriate than GPS‐established coordinates at the fine scale. Although we used ground beetles as a model group, our findings can be relevant for tracking any walking species with limited dispersal power, from ground‐dwelling arthropods to small vertebrates, such as frogs or lizards. The limitations in the precision of GPS units should be taken into account, especially in studies focused on microhabitat use. Although the distance‐bearing method can be more time‐consuming due to the manual record of each fix using a compass and a measuring tape, this method is more accurate and the derived movement parameters are more reliable.

## CONFLICT OF INTEREST

The authors declare that they have no conflict of interest.

## AUTHOR CONTRIBUTION

**Jana Růžičková:** Conceptualization (equal); Formal analysis (equal); Investigation (equal); Methodology (equal); Writing‐original draft (equal). **Zoltán Elek:** Conceptualization (equal); Formal analysis (equal); Investigation (equal); Methodology (equal); Writing‐original draft (equal).

## Supporting information

Fig S1

## Data Availability

Data are available from the Dryad Digital Repository at https://doi.org/10.5061/dryad.79cnp5hvj.

## References

[ece37670-bib-0001] Adrados, C. , Girard, I. , Gendner, J. P. , & Janeau, G. (2002). Global positioning system (GPS) location accuracy improvement due to selective availability removal. Comptes Rendus Biologies, 325, 165–170. 10.1016/S1631-0691(02)01414-2 11980177

[ece37670-bib-0002] Baars, M. A. (1979). Patterns of movement of radioactive carabid beetles. Oecologia, 44, 125–140. 10.1007/BF00346411 28310477

[ece37670-bib-0003] Bartoń, K. (2016). MuMIn: Multi‐model inference. https://CRAN.R‐project.org/package=MuMIn

[ece37670-bib-0004] Bates, D. , Mächler, M. , Bolker, B. , & Walker, S. (2015). Fitting linear mixed‐effects models using lme4. Journal of Statistical Software, 67, 1–48. 10.18637/jss.v067.i01

[ece37670-bib-0005] Batsleer, F. , Bonte, D. , Dekeukeleire, D. , Goossens, S. , Poelmans, W. , Van der Cruyssen, E. , Maes, D. , & Vandegehuchte, M. L. (2020). The neglected impact of tracking devices on terrestrial arthropods. Methods in Ecology and Evolution, 11, 350–361. 10.1111/2041-210X.13356

[ece37670-bib-0006] Bérces, S. , & Růžičková, J. (2019). Habitat use of an endangered beetle Carabus hungaricus assessed via radio telemetry. Acta Zoologica Academiae Scientiarum Hungaricae, 65, 335–348. 10.17109/AZH.65.4.335.2019

[ece37670-bib-0007] Bradshaw, C. J. , Sims, D. W. , & Hays, G. C. (2007). Measurement error causes scale‐dependent threshold erosion of biological signals in animal movement data. Ecological Applications, 17, 628–638. 10.1890/06-0964 17489266

[ece37670-bib-0008] Cagnacci, F. , Boitani, L. , Powell, R. A. , & Boyce, M. S. (2010). Animal ecology meets GPS‐based radiotelemetry: A perfect storm of opportunities and challenges. Philosophical Transactions of the Royal Society B, 365, 2157–2162. 10.1098/rstb.2010.0107 PMC2894970 20566493

[ece37670-bib-0009] Calenge, C. (2006). The package “adehabitat” for the R software: Tool for the analysis of space and habitat use by animals. Ecological Modelling, 197, 516–519. 10.1016/j.ecolmodel.2006.03.017

[ece37670-bib-0010] Calenge, C. , Dray, S. , & Royer‐Carenzi, M. (2009). The concept of animals' trajectories from a data analysis perspective. Ecological Informatics, 4, 34–41. 10.1016/j.ecoinf.2008.10.002

[ece37670-bib-0011] de Paulo, M. , Laplante, F. , Australia, D. M. S. , Technology, O. , & Petroff, M. (2016). Azimuth and distance plugin. https://github.com/mpetroff/qgsazimuth

[ece37670-bib-0012] D'Eon, R. G. , Serrouya, R. , Smith, G. , & Kochanny, C. O. (2002). GPS radiotelemetry error and bias in mountainous terrain. Wildlife Society Bulletin, 30, 430–439.

[ece37670-bib-0013] Dray, S. , Royer‐Carenzi, M. , & Calenge, C. (2010). The exploratory analysis of autocorrelation in animal‐movement studies. Ecological Research, 25, 673–681. 10.1007/s11284-010-0701-7

[ece37670-bib-0014] Elek, Z. , Kovács, B. , Aszalós, R. , Boros, G. , Samu, F. , Tinya, F. , & Ódor, P. (2018). Taxon‐specific responses to different forestry treatments in a temperate forest. Scientific Report, 8, 16990. 10.1038/s41598-018-35159-z PMC6243015 30451880

[ece37670-bib-0015] Elek, Z. , Růžičková, J. , & Ódor, P. (2019). Individual decisions drive the changes in movement patterns of ground beetles between forestry management types in Hungary. 2nd International Conference on Community Ecology, Book of abstracts (pp. 79). Akadémiai Kiadó. https://static.akcongress.com/downloads/comec/comec2019‐boa.pdf

[ece37670-bib-0016] Fernández, P. , Rodríguez, A. , Obregón, R. , de Haro, S. , Jordano, D. , & Fernández‐Haeger, J. (2016). Fine scale movements of the butterfly *Plebejus argus* in a heterogeneous natural landscape as revealed by GPS tracking. Journal of Insect Behavior, 29, 80–98. 10.1007/s10905-016-9543-7

[ece37670-bib-0017] Fisher, K. E. , Adelman, J. S. , & Bradbury, S. P. (2020). Employing very high frequency (VHF) radio telemetry to recreate monarch butterfly flight paths. Environmental Entomology, 49, 312–323. 10.1093/ee/nvaa019 32159219

[ece37670-bib-0018] Fisher, K. E. , Dixon, P. M. , Han, G. , Adelman, J. S. , & Bradbury, S. P. (2020). Locating large insects using automated VHF radio telemetry with a multi‐antennae array. Methods in Ecology and Evolution, 12(3), 494–506. 10.1111/2041-210X.13529

[ece37670-bib-0019] Frair, J. L. , Fieberg, J. , Hebblewhite, M. , Cagnacci, F. , DeCesare, N. J. , & Pedrotti, L. (2010). Resolving issues of imprecise and habitat‐biased locations in ecological analyses using GPS telemetry data. Philosophical Transactions of the Royal Society B, 365, 2187–2200. 10.1098/rstb.2010.0084 PMC2894963 20566496

[ece37670-bib-0020] Grueber, C. E. , Nakagawa, S. , Laws, R. J. , & Jamieson, I. G. (2011). Multimodel inference in ecology and evolution: Challenges and solutions. Journal of Evolutionary Biology, 24, 699–711. 10.1111/j.1420-9101.2010.02210.x 21272107

[ece37670-bib-0021] Holyoak, M. , Casagrandi, R. , Nathan, R. , Revilla, E. , & Spiegel, O. (2008). Trends and missing parts in the study of movement ecology. Proceedings of the National Academy of Sciences, 105, 19060–19065. 10.1073/pnas.0800483105 PMC2614715 19060194

[ece37670-bib-0022] Jerde, C. L. , & Visscher, D. R. (2005). GPS measurement error influences on movement model parameterization. Ecological Applications, 15, 806–810. 10.1890/04-0895

[ece37670-bib-0023] Kareiva, P. M. , & Shigesada, N. (1983). Analyzing insect movement as a correlated random walk. Oecologia, 56, 234–238. 10.1007/BF00379695 28310199

[ece37670-bib-0024] Marsh, L. M. , & Jones, R. E. (1988). The form and consequences of random walk movement models. Journal of Theoretical Biology, 133, 113–131. 10.1016/S0022-5193(88)80028-6

[ece37670-bib-0025] Michelot, T. , Langrock, R. , & Patterson, T. A. (2016). moveHMM: An R package for the statistical modelling of animal movement data using hidden Markov models. Methods in Ecology and Evolution, 7, 1308–1315. 10.1111/2041-210X.12578

[ece37670-bib-0026] Millspaugh, J. J. , & Marzluff, J. M. (2001). Radio tracking and animal populations. Academic Press.

[ece37670-bib-0027] Montgomery, R. A. , Roloff, G. J. , Hoef, J. M. V. , & Millspaugh, J. J. (2010). Can we accurately characterize wildlife resource use when telemetry data are imprecise? Journal of Wildlife Management, 74, 1917–1925. 10.2193/2010-019

[ece37670-bib-0028] Nathan, R. , Getz, W. M. , Revilla, E. , Holyoak, M. , Kadmon, R. , Saltz, D. , & Smouse, P. E. (2008). A movement ecology paradigm for unifying organismal movement research. Proceedings of the National Academy of Sciences, 105, 19052–19059. 10.1073/pnas.0800375105 PMC2614714 19060196

[ece37670-bib-0029] Negro, M. , Caprio, E. , Leo, K. , Maritano, U. , Roggero, A. , Vacchiano, G. , Palestrini, C. , & Rolando, A. (2017). The effect of forest management on endangered insects assessed by radio‐tracking: The case of the ground beetle *Carabus olympiae* in European beech *Fagus sylvatica* stands. Forest Ecology and Management, 406, 125–137. 10.1016/j.foreco.2017.09.065

[ece37670-bib-0030] Negro, M. , Casale, A. , Migliore, L. , Palestrini, C. , & Rolando, A. (2008). Habitat use and movement patterns in the endangered ground beetle species, *Carabus olympiae* (Coleoptera: Carabidae). European Journal of Entomology, 105, 105–112. 10.14411/eje.2008.015

[ece37670-bib-0031] Negro, M. , Vacchiano, G. , Berretti, R. , Chamberlain, D. E. , Palestrini, C. , Motta, R. , & Rolando, A. (2014). Effects of forest management on ground beetle diversity in alpine beech (*Fagus sylvatica* L.) stands. Forest Ecology and Management, 328, 300–309. 10.1016/j.foreco.2014.05.049

[ece37670-bib-0032] Niemelä, J. , Haila, Y. , Halme, E. , Pajunen, T. , & Punttila, P. (1992). Small‐scale heterogeneity in the spatial distribution of carabid beetles in the southern Finnish taiga. Journal of Biogeography, 19, 173–181. 10.2307/2845503

[ece37670-bib-0033] Pearce, J. L. , Venier, L. A. , McKee, J. , Pedlar, J. , & McKenney, D. (2003). Influence of habitat and microhabitat on carabid (Coleoptera: Carabidae) assemblages in four stand types. The Canadian Entomologist, 135, 337–357. 10.4039/n02-031

[ece37670-bib-0034] QGIS Development Team . (2019). QGIS Geographic Information System. Open Source Geospatial Foundation Project. http://qgis.osgeo.org

[ece37670-bib-0035] R Core Team (2019). R: A language and environment for statistical computing. R Foundation for Statistical Computing.

[ece37670-bib-0036] Ranacher, P. , Brunauer, R. , Trutschnig, W. , Van der Spek, S. , & Reich, S. (2016). Why GPS makes distances bigger than they are. International Journal of Geographical Information Science, 30, 316–333. 10.1080/13658816.2015.1086924 27019610 PMC4786863

[ece37670-bib-0037] Riecken, U. , & Raths, U. (1996). Use of radio telemetry for studying dispersal and habitat use of *Carabus coriaceus* L. Annales Zoologici Fennici, 33, 109–116.

[ece37670-bib-0038] Rink, M. , & Sinsch, U. (2007). Radio‐telemetric monitoring of dispersing stag beetles: Implications for conservation. Journal of Zoology, 272, 235–243. 10.1111/j.1469-7998.2006.00282.x

[ece37670-bib-0039] Robinson, S. G. , Weithman, C. E. , Bellman, H. A. , Prisley, S. P. , Fraser, J. D. , Catlin, D. H. , & Karpanty, S. M. (2020). Assessing error in locations of conspicuous wildlife using handheld GPS units and location offset methods. Wildlife Society Bulletin, 44, 163–172. 10.1002/wsb.1055

[ece37670-bib-0040] Růžičková, J. , & Veselý, M. (2016). Using radio telemetry to track ground beetles: Movement of *Carabus ullrichii* . Biologia, 71, 924–930. 10.1515/biolog-2016-0108

[ece37670-bib-0041] Růžičková, J. , & Veselý, M. (2018). Movement activity and habitat use of *Carabus ullrichii* (Coleoptera: Carabidae): The forest edge as a mating site? Entomological Science, 21, 76–83. 10.1111/ens.12286

[ece37670-bib-0042] Schultz, C. B. , & Crone, E. E. (2001). Edge‐mediated dispersal behavior in a prairie butterfly. Ecology, 82, 1879–1892. 10.2307/2680054

[ece37670-bib-0043] Skórka, P. , Nowicki, P. , Lenda, M. , Witek, M. , Śliwińska, E. B. , Settele, J. , & Woyciechowski, M. (2013). Different flight behaviour of the endangered scarce large blue butterfly *Phengaris teleius* (Lepidoptera: Lycaenidae) within and outside its habitat patches. Landscape Ecology, 28, 533–546. 10.1007/s10980-013-9855-3

[ece37670-bib-0044] Turchin, P. , Odendaal, F. J. , & Rausher, M. D. (1991). Quantifying insect movement in the field. Environmental Entomology, 20, 955–963. 10.1093/ee/20.4.955

[ece37670-bib-0045] Wallin, H. , & Ekbom, B. S. (1988). Movements of carabid beetles (Coleoptera: Carabidae) inhabiting cereal fields: A field tracing study. Oecologia, 77, 39–43. 10.1007/BF00380922 28312312

[ece37670-bib-0046] Wehnert, A. , & Wagner, S. (2019). Niche partitioning in carabids: Single‐tree admixtures matter. Insect Conservation and Diversity, 12, 131–146. 10.1111/icad.12321

[ece37670-bib-0047] White, G. C. , & Garrott, R. A. (1990). Analysis of wildlife radio‐tracking data. Academic Press.

