# Supplementary material for: Recording fine‐scale movement of ground beetles by two methods: Potentials and methodological pitfalls
Source: Ecol Evol. 2021 May 16;11(13):8562–72. doi: 10.1002/ece3.7670 (PMC8258227; doi:10.1002/ece3.7670)

**Online supplementary material**

Růžičková and Elek: *Recording fine-scale movement of ground beetles by two methods: potentials and methodological pitfalls*

**ESM Figure 1:** DB-measured trajectories (track ID ends on “a”; on the left) and their GPS-measured counterparts (b-d, each of three builds is plotted separately) colored by movement states (orange: random walk, blue: directed movement) based on hidden Markov models. X-coordinates are for longitude (°E), y-coordinates for latitude (°N) respectively.


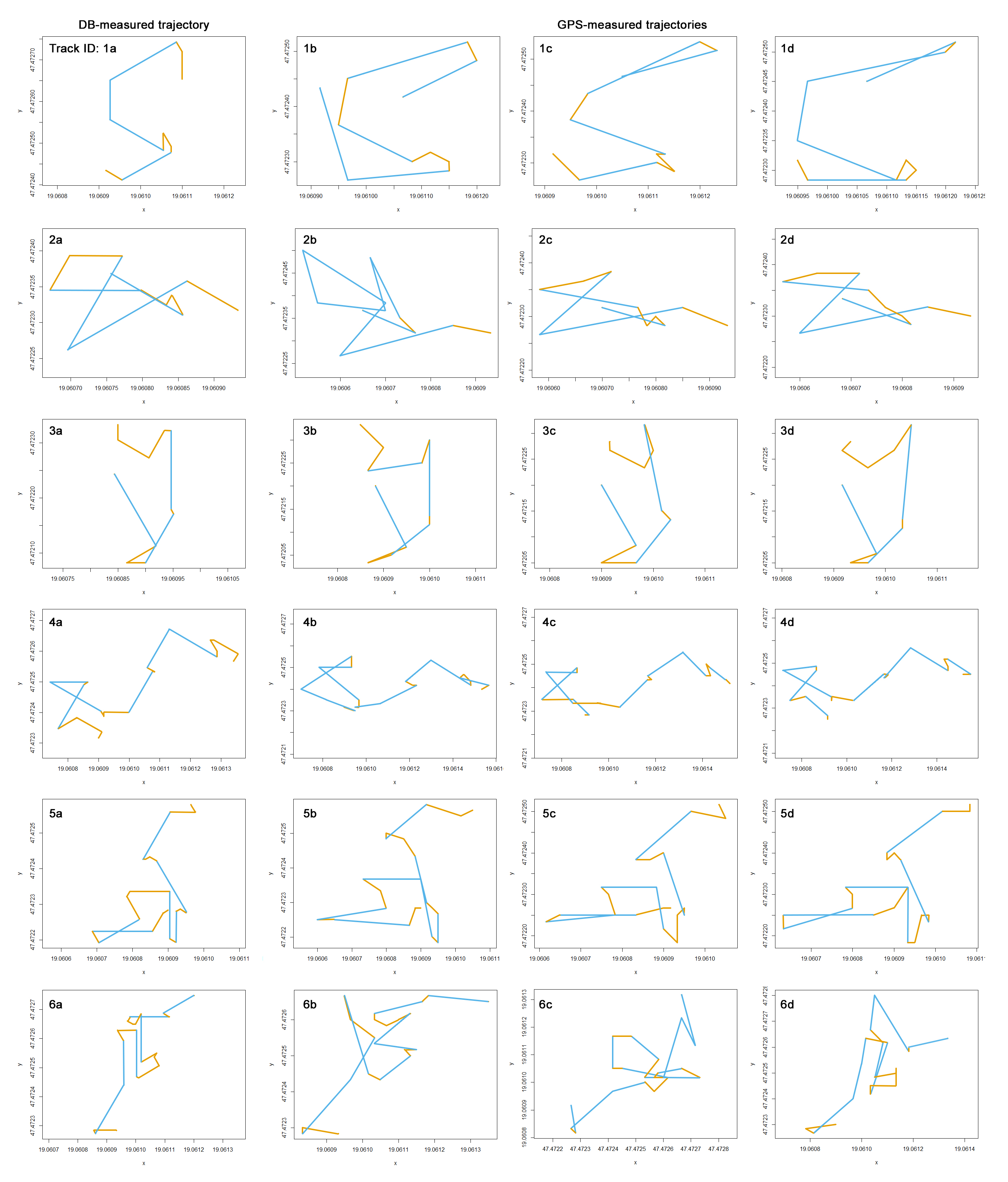

Supplement: Supplementary file 1 — Fig S1 [file ECE3-11-8562-s001.doc]
